# Uniform Orientation of Biotinylated Nanobody as an Affinity Binder for Detection of *Bacillus thuringiensis* (Bt) Cry1Ac Toxin

**DOI:** 10.3390/toxins6123208

**Published:** 2014-12-02

**Authors:** Min Li, Min Zhu, Cunzheng Zhang, Xianjin Liu, Yakun Wan

**Affiliations:** 1 The Key Laboratory of Developmental Genes and Human Disease, Ministry of Education, Institute of Life Sciences, Sipailou NO. 2, Southeast University, Nanjing 210096, China; E-Mails: damin.li@163.com (M.L.); zhumin555111@163.com (M.Z.); 2 Institute of Food Safety, Jiangsu Academy of Agricultural Sciences, Nanjing 210014, China; E-Mail: zhcz2003@hotmail.com; 3 Jiangsu Nanobody Engineering and Research Center, Nantong 226010, China

**Keywords:** nanobody, Cry1Ac toxin, streptavidin, DAS-ELISA

## Abstract

Nanobodies are the smallest natural fragments with useful properties such as high affinity, distinct paratope and high stability, which make them an ideal tool for detecting target antigens. In this study, we generated and characterized nanobodies against the Cry1Ac toxin and applied them in a biotin-streptavidin based double antibodies (nanobodies) sandwich-ELISA (DAS-ELISA) assay. After immunizing a camel with soluble Cry1Ac toxin, a phage displayed library was constructed to generate Nbs against the Cry1Ac toxin. Through successive rounds of affinity bio-panning, four nanobodies with greatest diversity in CDR3 sequences were obtained. After affinity determination and conjugating to HRP, two nanobodies with high affinity which can recognize different epitopes of the same antigen (Cry1Ac) were selected as capture antibody (Nb61) and detection antibody (Nb44). The capture antibody (Nb61) was biotinylated *in vivo* for directional immobilization on wells coated with streptavidin matrix. Both results of specificity analysis and thermal stability determination add support for reliability of the following DAS-ELISA with a minimum detection limit of 0.005 μg·mL^−1^ and a working range 0.010–1.0 μg·mL^−1^. The linear curve displayed an acceptable correlation coefficient of 0.9976. These results indicated promising applications of nanobodies for detection of Cry1Ac toxin with biotin-streptavidin based DAS-ELISA system.

## 1. Introduction

Bt toxins are known as insecticidal crystal proteins produced by Gram-positive spore-forming soil bacterium *Bacillus thuringiensis* [1]. They have a widespread use in agriculture for the control of insect pests as spray insecticides or expressed in genetically modified (GM) crops because of their high effectiveness and specificity to target insects [2,3]. Crystalline (Cry) toxins are the most widely used Bt proteins, particularly Cry1Ab in Bt corn and Cry1Ac in Bt cotton that kill the lepidopteran larvae [4,5,6]. However, concerns regarding the potential risks have been raised because of food and environmental safety questions as well as social and economic issues caused by GM crops since commercialized Bt products were introduced decades ago [7,8,9]. Previous research has demonstrated that GM crops changed nutrition-related properties [10]. Others even reported that Cry proteins have caused hematotoxicity in mice [11]. It is envisaged that both the potential broad applications and risks of Cry toxins will continue to draw the attention of the public, which brings the purpose of the study which is of vital importance: to establish an effective and rapid method for detecting insecticidal Cry1Ac toxin sensitively.

To detect GM crops and their products, numerous methods have been developed primarily based on DNA and proteins [12], such as the polymerase chain reaction (PCR) [13], real-time PCR, enzyme-linked immunosorbent assay (ELISA) and immuno-strip [14,15]. PCR and real-time PCR are commonly applied approaches for qualitative and quantitative detection of Cry genes, which require people to know the sequence of target genes for designing a primer for amplification [16]. Sensitive and specific as the above methods are, they are not adequate for extensive detecting because identifying the expression level of Cry toxin proteins is difficult to achieve without other methods [17]. One of the currently described immunoassay techniques based on antibodies for the detection of transgenic proteins is enzyme-linked immunosorbent assay (ELISA) [15], which as an alternative, has significant advantages for protein detection. However, it is difficult and tedious to obtain antibodies with high affinity and specificity, which is usually used in a sandwich ELISA for binding target Bt Cry toxin [15]. Therefore, it is highly desirable to develop rapid and innovative detection methods of Cry toxins.

As a result of the development of bioengineering technology, single-domain antigen-binding fragments, known as VHHs or nanobodies consist of about 120 amino acids [18,19] and have been widely applicable and complementary to other protein scaffolds and antibody formats [20]. They are the smallest natural fragments which have been identified to date [19,21], and their introduction in various applications has been stimulated by their beneficial properties such as high affinity, distinct paratope and small size [20]. In addition to their high thermal stability, nanobodies are more soluble and less immunogenic than other antibody fragments [22]. All these peculiar properties make nanobodies an ideal tool for the detection of some target antigens with cryptic, conformational epitopes that cannot be reached and recognized by conventional antibodies [23]. Nanobodies have been successfully used for detection and analysis purposes, such as nanobody-based detection of human procalcitoninand human prealbumin which belong to the previous works of our team [24,25], and nanobody-based system for cell-specific gene manipulation developed by another team [26].

In this study, we firstly generated and characterized nanobodies with high affinity, specificity and stability against the Cry1Ac of Bt toxins by using phage display technology and other analytical methods. The expressed anti-Cry1Ac nanobodies were purified and modified so as to be applied in a DAS-ELISA assay. This system showed a promising future in detection of Cry1Ac toxin.

## 2. Results and Discussion

### 2.1. Immunized VHH Library Construction

The single domain antibody library expected to have high affinity and specificity was constructed after immunization of a healthy camel with weekly injections of Cry1Ac. The stepwise PCR which assured the feasibility and accuracy in the whole constructing process was performed. PCR products for VHH-CH2-CH3 exons including ~700 bp (Figure S1A) were amplified and used as template for second PCR (Figure S1B) from which ~400 bp VHH fragments were obtained and amplified. The library was estimated to be around 1 × 10^9^ colonies, with an insertion rate of 100% according to PCR analysis by randomly choosing 24 colonies (Figure S1C). These results indicated that an immunized phage display library with high quality was successfully constructed for the following Cry1Ac-specific nanobody selection.

### 2.2. Library Screening and Identification of Cry1Ac-Specific Nanobodies

Bio-panning based on phage display was performed to identify the Cry1Ac-specific nanobodies from a phage display library constructed with high quality. In order to make sure the antigen of Cry1Ac was pure enough in the following bio-panning, the purity was firstly checked by SDS-PAGE and it showed great purity (Figure S2A). Approximately 2 × 10^11^ phages from the library were used for panning. The phages expressing Cry1Ac-specific VHHs were enriched by consecutive rounds of bio-panning on Cry1Ac. Compared to the negative control, the enrichment fold of specific VHHs reached to 200, sharply raised after two rounds of panning (Figure S2B), revealing that the majority of specific VHHs could be selected from second rounds [24,25,27].

**Figure 1:**
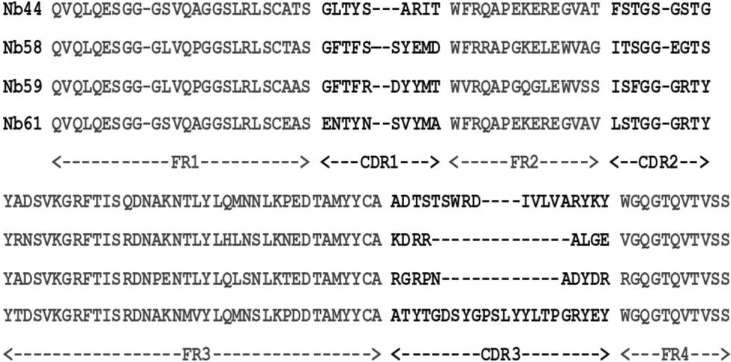
Four classes of different amino acid sequences of anti-Cry1Ac VHHs were identified after the VHH genes against Cry 1Ac were selected by phage display library.

To obtain positive clones, 95 individual clones were chosen randomly from rounds of panning for Periplasmic extract ELISA (PE-ELISA). This procedure is based on the principle that nanobodies resided in supernatant of the cells disrupted by osmotic shock can bind to the corresponding antigen directly [24,25]. 19 colonies were selected as positive clones whose ratio to control of absorbance at 405 nm was more than 2 (Figure S2C), which meant that these clones contained nanobodies which can specifically bind to Cry1Ac. After performing PE-ELISA, the positive colonies were sequenced and analyzed. The selected VHHs can be categorized into 4 groups (Nb44, Nb58, Nb59 and Nb61) according to the diversity of their amino acid sequences in CDR3, which is the main contributor of antigen binding (Figure 1) [28].

### 2.3. Nbs Expression, Purification and Affinity Determination

**Figure 2:**
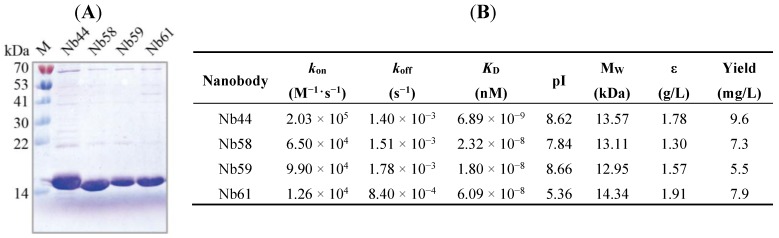
(**A**) SDS-PAGE analysis of purified Cry1Ac-specific nanobodies by coomassie staining; (**B**) Properties of Cry1Ac-specific nanobodies. The affinity of these nanobodies was determined by Biacore. Molecular mass and theoretical pI were calculated using the ExPASy ProtParam Tool (http://web.expasy.org/protparam/).

Generally, VHH fragments encoding the four anti-Cry1Ac nanobodies (Nb44, Nb58, Nb59 and Nb61) were transformed into *E. coli* WK6 electrocompetent cells respectively from TG1 strain by conventional method [27]. In this study, the inducible periplasmic expression of Nbs as soluble *C*-terminally His_6_-tagged proteins was permitted by display vector pMECS. WK6 strain has the ability to suppress the amber stop codon between gene III and VHH gene on pMECS while TG1 does not have the property, which is one of the reasons why the recombinant phagemid need to be transformed from TG1 to WK6 strain without doing subcloning step. SDS-PAGE analysis were performed after purifying soluble Nbs with NI-NTA Superflow Sepharose columns, which showed a band corresponding approximately to 15 kD molecular weight (Figure 2A), demonstrating that high quality of Nbs with the purity of more than 90% were successfully expressed and purified.

As the result of kinetic analysis by Surface Plasmon Resonance (SPR) experiment presented, three of the anti-Cry1Ac Nbs showed weak alkalinity with theoretical isoelectric point (pI) at 7.84–8.66 and one (Nb61) possessed weak acidity with pI at 5.5 (Figure 2B). The affinities of the 4 selected Nbs against Cry1Ac molecules were measured and as shown in Figure 2B, Nbs presented high affinities with *K_D_* ranging from 1.80 × 10^−8^ to 6.89 × 10^−9^ nM (Figure 2B). To sum up, these four anti-Cry1Ac Nbs (Nb44, Nb58, Nb59 and Nb61) showed high affinities which meant strong binding ability to Cry1Ac and would lead to its strong applied value in areas such as detection and diagnosis [24,25].

### 2.4. Nanobody Conjugation to HRP in Vitro and Biotinylation in Vivo

Nanobodies were conjugated to horseradish peroxidase (HRP) as signal indicators in color reactions. On the other hand, by taking advantage of these Nb-HRPs, it can be known whether Nbs recognize different epitopes of the same antigen (Cry1Ac). After performing this process, 2 nanobodies (Nb44 and Nb61) against Cry1Ac with high affinity were selected as capture antibody (Nb44) and detection antibody (Nb61), respectively.

In this study, the capture antibody was immobilized on wells coated with streptavidin matrix for capturing Cry1Ac in corresponding reagent. Thus, Nb44, with the highest affinity (*K_D_* = 6.89 × 10^−9^ nM), was selected to be labeled with Biotin *in vivo*. In order to obtain biotinylated nanobody, VHH genes were sub-cloned into plasmid pBAD which contained a Biotin Acceptor Domain (BAD) and then were co-transformed into WK6 cells with plasmid BirA (encoding biotin protein ligase) for the expression of biotin-conjugating Nb (Cry1Ac Nb44-Biotin) which were further purified with Streptavidin-Mutein Matrix and it was produced at a final yield of 1.0 mg·L^−1^ culture.

### 2.5. Specificity and Thermostability Analysis

**Figure 3:**
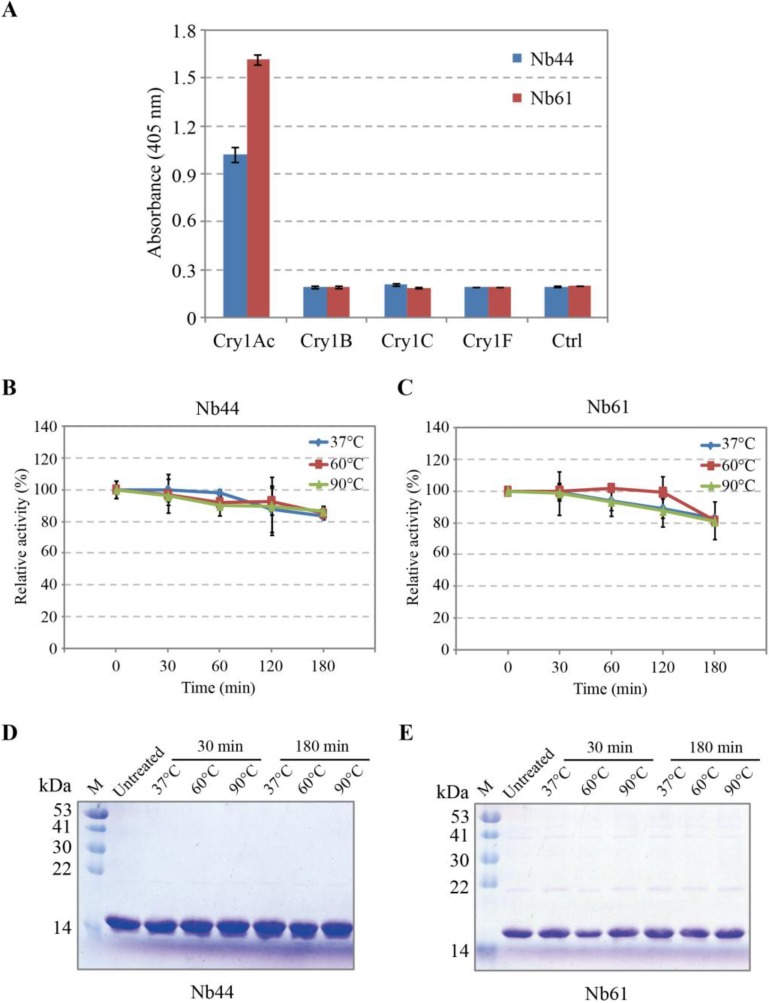
Specificity and stability detection of paired nanobodies (**A**) Specificity detection of soluble and purified Nb44 and Nb61 by ELISA. Four members of Cry1 family (Cry1Ac, Cry1B, Cry1C, Cry1F) were coated onto microtiter plates and nanobodies were added to incubate with them. After reaction with mouse anti-HA tag antibody and then anti-mouse IgG-alkaline phosphatase, the chromogenic solution containing Bis phosphate was added and the absorbance at 405 nm were measured by an ELISA reader. Values were the means of three replicates; (**B**,**C**) Stability detection of nanobodies by ELISA after treated at different temperatures for several hours. These nanobodies were put at 37 °C, 60 °C, 90 °C for 30, 60, 120, 180 min respectively, then incubated with Cry1Ac. The relative activity of untreated samples was considered as 100%. The results were from triplicate measurements; (**D**,**E**) SDS-PAGE analysis of nanobodies treated for different time.

The specificity of the proposed nanobodies (Nb44 and Nb61) was examined by ELISA (Figure 3A). The results showed that both Nb44 and Nb61 had strongly binding ability to Cry1Ac toxin, but not to Cry1B, Cry1C and Cry1F toxin.

As shown in Figure 3B,C, after treatment for different hours at different temperature, both the activity of Nb44 and Nb61 showed slight and inconspicuous downward trends. Even under treatment for 3 h at the temperature of 90 °C, Nb44 still kept nearly 85% activity and Nb61 kept nearly 82% activity. Apparently, these data inferred that both Nb44 and Nb61 showed good thermal stability and their useful properties make them become promising detection and analysis tools for various purposes in severe environments. Meanwhile, according to Figure 3D,E of the results of SDS-PAGE, it can be estimated that these nanobodies did not degrade, which inferred that the gradual decreased signals upon incubation at 37 °C, 60 °C and 90 °C are due to the decreasing activity of nanobodies.

### 2.6. Cry1Ac Toxin Detection by DAS-ELISA

**Scheme 1:**
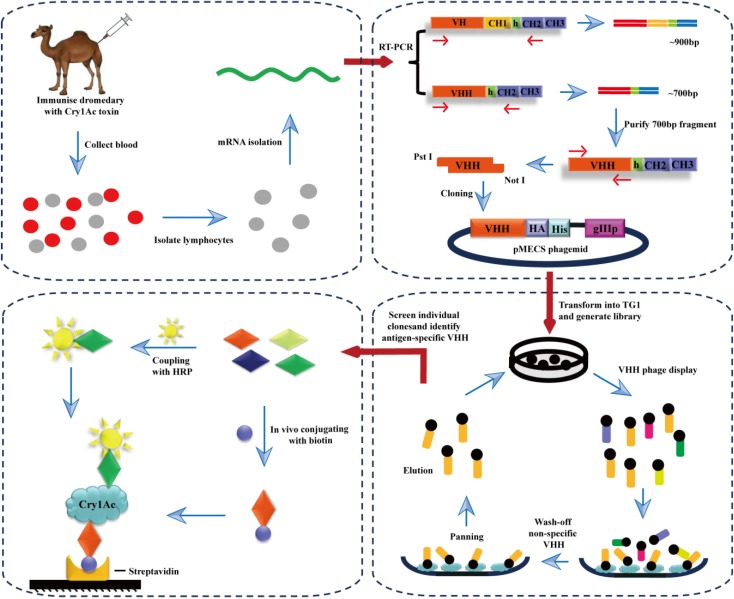
Outline of strategies to select the Cry1Ac-specific nanobodies from an immunized camel and to develop a directional system of double nanobodies sandwich ELISA.

As shown in Scheme 1, biotinylated nanobodies (Nb44-Biotin) were directionally captured by the streptavidin matrix coated on the plate, since the non-covalent interaction between biotin and streptavidin was strong and hard to break. After Cry1Ac was recognized and captured by biotinylated nanoboies, nanobodies coupling with HRP (Nb61-HRP) were applied in the downstream detection.

**Figure 4:**
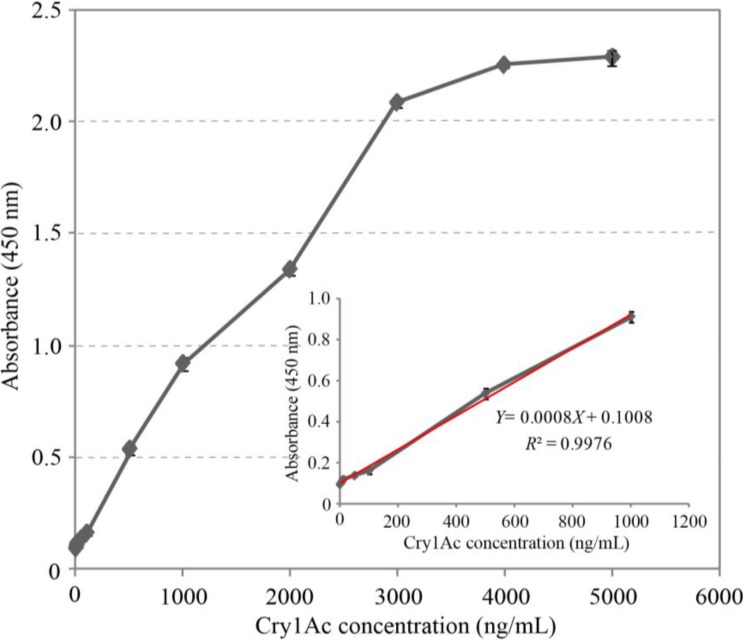
Detection of Cry1Ac toxin by sandwich ELISA. Serial concentrations (0, 10, 50, 100, 500, 1000, 5000 ng·mL^−1^) of Cry1Ac toxin were used for detection. The linear relationship was in the range from 10 to 1000 ng·mL^−1^. The linear equation was calculated as *Y* = 0.0008 *X* + 0.1008 with an acceptable correlation coefficient of 0.9976 (*R*^2^). The data represent mean ± standard deviation from triplicate measurements.

The result of this streptavidin-biotin-based directional double antibody (nanobodies) sandwich ELISA was shown in Figure 4. The absorbance at 450 nm displayed a good linearity with the concentrations of Cry1Ac ranging from 0.010 to 1.0 g·mL^−1^. The linear equation was calculated as (1) with an acceptable correlation coefficient of 0.9976 (*R*^2^), and the minimum detection limit (calculated according to the published method in [29]) was 0.005 μg·mL^−1^, illustrating good sensitivity and strong application value of this method for Cry1Ac detection.

(1) Y=0.0008 X+0.1008

**Table 1:** Recoveries of Cry1Ac toxin from spiked samples by sandwich ELISA.

Sample		Cry1Ac Concentration (ng/mL)		Recovery (%)	RSD (%)
		Added	Found		
Corn	1	50	40.66	81.32	4.32
	2	100	82.68	82.68	7.09
	3	250	275.02	110.00	6.83
	4	500	424.80	86.96	4.70
	5	1000	1024.40	104.24	5.12
Soybean	1	50	48.29	96.59	5.19
	2	100	106.19	106.19	7.36
	3	250	272.18	107.21	6.35
	4	500	420.80	84.16	2.52
	5	1000	1029.60	102.96	3.28

Data from ELISA represent the means of triplicate measurements. The coefficient of variation (CV, RSD) is calculated from the average and standard deviation as follows: (Standard Deviation/Average) × 100%.

### 2.7. Assessment of the DAS-ELISA by Spiked Samples

The measured recoveries of Cry1Ac from the spiked samples are shown in Table 1. The average recoveries ranged from 81.32% to 110.0%, with a coefficient of variation (C.V) between 2.52% and 7.36%, theoretically and practically showing excellent sensitivity and accuracy for the quantitative detection of Cry1Ac toxin based on the directional system of double nanobodies sandwich ELISA in environmental and agricultural samples.

## 3. Experimental Section

### 3.1. Chemicals and Materials

Cry toxins this study used were purchased from YouLong Bio. Co. Ltd. (Shanghai, China) and dissolved in 1 × PBS, stored at −80 °C. Freund’s complete adjuvant and Freund’s incomplete adjuvant were purchased from Sigma-Aldrich (Shanghai, China). Fast Track 2.0 Kit was provided by Invitrogen (Carsbad, CA, USA) and Oligo_dT_ primer was obtained from Thermo Scientific (Waltham, MA, USA). Restriction enzymes *Pst* I, *Not* I, *Nco* I and *BstE* II were provided from NEB (Ipswich, MA, USA). Mouse anti-HA tag antibody was purchased from Covance (Princeton, NJ, USA). Anti-mouse IgG-alkaline phosphatase, Bis phosphate and His-Select column were purchased from Sigma-aldrich (Shanghai, China). D-biotin and horseradish peroxidase (HRP) were purchased from Bio Basic Inc. (Shanghai, China). Streptavidin Mutein Matrix was purchased from Roche (Basel, Switzerland). Microtitre plate was purchased from Thermo Scientific NUNC (Roskilde, Denmark). BeaverNano™ Streptavidin Matrix Coated 96-Well plates were provided by BeaverNano technology company (Suzhou, China). VCSM13 helper phages, TG1 cells, WK6 cells, plasmid pBAD and plasmid pBirA were obtained from Prof. Serge Muyldermans’s lab (Laboratory of Cellular and Molecular Immunology, Vrije Universiteit Brussel, Brussels, Belgium). All other reagents were of analytical grade and used as received.

### 3.2. Dromedary Immunization

A healthy young dromedary camel was immunized by weekly injections of soluble and pure antigen (Cry1Ac) (1 mL, 1 mg) mixed with Freund’s incomplete adjuvant, to stimulate antigen-specific B cells to express nanobodies. In addition, after 6 times, lymphocytes were extracted from 100 mL peripheral blood collected from the dromedary for constructing the library.

### 3.3. Library Construction

PBLs (Peripheral blood lymphocytes) were isolated from an immunized dromedary camel, by density gradient centrifugation with Ficoll-paqueTM plus (GE healthcare, Beijing, China). Then by using Fast Track 2.0 Kit (Invitrogen, Carsbad, CA, USA), total RNA was extracted from the purified PBLs and its concentration was calculated then. The variable regions of heavy-chain immunoglobulins (VHH) were amplified by nested PCR for the purpose of avoiding contaminating with VH genes. PCR conditions were: 1 cycle at 94 °C for 7 min, 30 cycles at 94 °C for 1 min, 55 °C for 1 min, 72 °C for 1 min and finally 1 cycle of 72 °C for 10 min. The secondary PCR amplification was done with templates near 700 base pairs (bp) from the first PCR products and used the same protocol as first PCR but reduced to 17 cycles with primers contained restriction sites *Pst* I and *Not* I. After being digested by *Pst* I and *Not* I restriction enzymes (NEB, Ipswich, MA, USA), the amplified second PCR products were unidirectionally ligated into *Pst* I-, *Not* I- and *Xba* I-digested pMECS to recover pMECS-VHH [30]. Then, the vector was electroporated into *Escherichia coli* TG1 electrocompetent cells at 1.7 kV followed by addition of 1 mL SOC media and shaking at 37 °C for 1 h. Finally, the library was stored in 15% glycerol at −80 °C until use [27]. Meanwhile, library size was evaluated by plating pooled transformants on LA selective medium (100 mg·Ampicillin/L), and randomly collected colonies were used for miniprep and detecting the insertion rate of library by PCR.

### 3.4. Selection of Cry1Ac-Specific Nanobodies from Phage-Display Library

VCSM13 was used as helper phages to infect a representative fraction of the VHH library cultured for bio-panning [30]. Cry1Ac toxin (the first round was 200 mg·mL^−1^, the second round was 100 mg·mL^−1^, respectively) in coating buffer (0.1 M NaHCO_3_, pH 8.2) was used as antigen to coat onto microtiter plates of 96-wells at 4 °C overnight, with 0.1 M NaHCO_3_ (pH 8.2) used as control. Wells were incubated with phages of the displayed library with PBS for 1 h at room temperature after blocking with 0.1% casein in phosphate-buffered saline (PBS) for 2 h. The binding phages were eluted with 100 mM triethylamine for 10 min and then immediately mixed with 1.0 M Tris-HCl (pH 7.4) for the purpose of neutralizing. Fresh exponentially growing culture of *E. coli* TG1 cells (OD_600_ = 0.4–0.6) were infected with the eluted phages and incubated at 37 °C in constant temperature incubator (without shaking) for 30 min to allow for optimal infection [17]. Phage particles were amplified for further rounds of bio-panning, following VCSM13 helper phage rescue.

The process above represented one round of bio-panning and after 2–4 rounds, the Cry1Ac-specific phages enriched.

### 3.5. Periplasmic Extract ELISA (PE-ELISA)

PE-ELISA is based on the principle that nanobodies resided in supernatant of the cells disrupted by osmotic shock and can bind to corresponding antigen directly. To obtain positive clones, 95 individual clones were picked randomly from rounds of panning for PE-ELISA. Each of these picked clones were cultured in 1 mL Terrific Broth (1L TB: 12 g peptone, 24 g yeast extract, 4 mL glycerol, 170 mM KH_2_PO_4_ and 0.72 M K_2_HPO_4_) containing 100 μg·mL^−1^ ampicillin. After 3 h of culturing, IPTG (1 mM) was added to induce the expression of nanobodies during overnight growing at 28 °C. Then cells collected were resuspended into 200 μL TES (0.5 M sucrose, 0.2 M Tris-HCl pH 8.0, 0.5 mM EDTA) for shaking 2 h at 4 °C, after which 300 μL TES/4 were added for shaking another 2 h at 4 °C. Next, the supernatant was collected and added into the plate wells which were coated with Cry1Ac toxin (2 μg·mL^−1^) in advance and for 1 h incubating. Then mouse anti-HA tag antibody and anti-mouse IgG-alkaline phosphatase were added for 1 h incubating successively. Finally, chromogenic solution containing Bis phosphate (pNPP) was added and after minutes of waiting the absorbance in 405 nm can be read with an ELISA reader (Bio-rad imark™, Hercules, CA, USA).

### 3.6. Expression and Purification of Soluble Nanobodies against Cry1Ac

Nanobodies were expressed by transforming the selected VHH genes in pMECS into *E. coli* WK6 electrocompetent cells, and these cells were grown in Terrific Broth containing 0.1% glucose, ampicillin (100 μg·mL^−1^) and 2 mM MgCl_2_. Expression of the nanobodies was induced subsequently by adding IPTG to a final concentration of 1 mM when the optical density (OD) was between 0.6 and 0.9 at 600 nm, and cultures were further grown for 16 h with shaking (220 rpm) at 28 °C. Then the cells collected from the 330 mL overnight culture were resuspended into 4 mL TES (0.5 M sucrose, 0.2 M Tris-HCl pH 8.0, 0.5 mM EDTA) for shaking 2 h at 4 °C, after which 8 mL TES/4 were added for shaking another 2 h at 4 °C. The supernatant containing the periplasmic proteins was collected and using His-Select columns (Sigma Aldrich, St. Louis, MO, USA), soluble Nb with His-tags were purified by Immobilized Metal Affinity Chromatography (IMAC). His-tagged proteins were eluted with gradient concentration of imidazole (pH 7.0) after washing 5 times with PBS. The purity of the eluted proteins was checked by 15% SDS-PAGE.

### 3.7. Affinity Determination

The affinities of selected anti-Cry1Ac Nbs were measured by the kinetic analysis by Surface Plasmon Resonance (SPR) experiment with PlexArray^®^ HT system (Plexera® Bioscience, Beijing, China) as previously described [24,25]. Firstly, 10 -Cry1Ac Nbs (10 μg·mL^−1^) were immobilized on PlexArray^®^ Nanocapture^®^ Sensor Chips (Plexera^®^ Bioscience, Beijing, China). Then, to take note of the signals, different concentrations of (0.12, 0.37, 1.1, 3.3 and 10 μg·mL^−1^) were injected with a flow rate of 3.0 μL·s^−1^. Finally, a regeneration step was performed after the affinity binding reaction. Briefly, the sensor chips were regenerated with 0.05 mol·L^−1^ NaOH for 2 min, and then PBS buffer was injected over the sensor surface for about 5 min. All signals were recorded as sensorgrams, and steps were performed in triplicate at 25 °C.

### 3.8. HRP Coupling: Preparation of HRP-Conjugated Second Antibody for Detection

The purified anti-Cry1Ac Nbs were conjugated to HRP as follows: 100 μL fresh NaIO_4_ (0.1 M) was mixed with 200 μL horseradish peroxidase (HRP) (Sigma-aldrich, St. Louis, MO, USA) (5 mg/mL) for 30 min incubation at 4 °C, after which an equal volume of ethylene glycol (2.5%) were added and incubated for 30 min at room temperature. Then 1 mL Cry1Ac-specific nanobodies (1 mg·L^−1^) were added, and after an overnight incubation at 4 °C in the dark, 20 μL Sodium borohydride (5 mg·mL^−1^) were mixed into it for 3 h at 4 °C and dialyzed into PBS. Nanobodies failed to be conjugated to HRP were removed by ultrafiltration and free HRP were eliminated by saturated ammonium sulfate. Generally, ammonium sulfate precipitation was started by adding the same volume of saturated ammonium sulfate to the mixture dropwise, while mixing it gently. After 1 h at 4 °C, the mixture was centrifuged at 3000 rpm for 30 min and then the precipitate was washed twice by using 50% saturated ammonium sulfate and dissolved in 0.15 M PBS (pH 7.4). Then the solution was dialyzed into PBS (pH 7.4) for the purpose of eliminating ammonium ion, after which the supernatants (namely Nb-HRPs) of the mixture were collected by centrifugation.

### 3.9. Nanobody Biotinylation: Preparation of Biotinylated First Antibody for Capture

Genes encoding anti-Cry1Ac VHHs were sub-cloned into plasmid pBAD by using *Nco* I and *BstE* II as restriction sites and the recombinant plasmid was co-transfected into WK6 competent cells with plasmid pBirA. 50 μM of d-biotin were added into medium 30 min before adding 1 mM IPTG to induce expression of VHH-BAD fusion proteins. By osmotic shock protocol, periplasmic proteins were extracted. Streptavidin Mutein Matrix was used for purifying biotinylated nanobodies (BiNb) eluted with 6 mM D-biotin solution.

### 3.10. ELISA for Nanobody Specificity Detection

In order to characterize the specificity of purified Nb44 and Nb61, 4 kinds of different Cry1 toxins (Cry1Ac, Cry1B, Cry1C and Cry1F) were chosen for ELISA. Firstly, 5 μg·mL^−1^ different Cry1 toxins in 0.1 M NaHCO_3_ (pH 8.2) were coated on plate wells overnight at 4 °C respectively, blocked with 200 mL per well of 1% BSA at room temperature for 2 h. Then Cry1Ac Nb44 and Nb61 (10 μg·mL^−1^) were added into the plate wells at room temperature for 1 h incubating. Following steps were undertaken as described in PE-ELISA.

### 3.11. Thermostability Analysis

To determine the thermal stability, different samples (Nb44 and Nb61) under different conditions were used for performing ELISA. The activity of Nb44 and Nb61 stored at −20 °C was regarded as 100%. Specifically, Cry1Ac Nb44 and Nb61 diluted in PBS was incubated at 37 °C, 60 °C and 90 °C respectively for 1 h, 2 h and 3 h with the no-treatment group of nanobodies stored at −20 °C being used as control, after which they were introduced to an ELISA plate coated with 5 μg·mL^−1^ of Cry1 Ac toxin. NaHCO_3_ (0.1 M, pH 8.2) was the blank control of each sample. 100 μL (2 μg·mL^−1^) of Cry1Ac Nb44 and Nb61 solution were added into corresponding wells to incubate for 1 h after the ELISA plate was blocked with 1% skim milk for 2 h. The rest of the experiments were the same as that described in PE-ELISA and ELISA for nanobody specificity detection. Meanwhile, these nanobodies (1 mg·mL^−1^) exposed to different conditions for different length of time were checked by 15% SDS-PAGE.

### 3.12. Cry1Ac Toxin Detection by DAS-ELISA Assay Based on Nbs and Directional System

Based on streptavidin-biotin system, directional double antibodies (nanobodies) sandwich ELISA was performed. 1 μg·mL^−1^ of biotinylated nanobody Cry1Ac Nb61 was added into plate coated with streptavidin matrix and incubated at room temperature for 1 h. Then different concentration gradients of Cry1Ac toxin (0, 10, 50, 100, 500, 1000, 5000 ng·mL^−1^) were added for 2 h after blocking the plate with 5% BSA for 1 h. Cry1Ac Nb44 conjugated with HRP were diluted with 5% BSA to 1 μg·mL^−1^ and incubated with Cry1Ac toxin for 1 h. Finally, the chromogenic reaction was measured by automatic micro-plate reader at 450 nm.

### 3.13. Assessment of the DAS-ELISA by Spiked Samples

The new DAS-ELISA method was assessed by performing Cry1Ac toxin determination in corn samples and soybean samples. 1 g of corn samples and soybean samples were spiked with Cry1Ac toxin at five concentration levels (0.05, 0.1, 0.25, 0.5 and 1.0 mg·kg^−1^), and then shaken with 1 mL of protein extraction solution containing 0.1 M PBS pH 7.4, containing 0.1% BSA and 0.05% Tween-20. The suspension was centrifuged for 15 min at 10,000 *g* after shaking at room temperature for 4 h [17]. Then the extract was diluted by 10-fold PBS and finally analyzed by DAS-ELISA. In all cases, standards and blanks (free of toxin samples) were used.

## 4. Conclusions

In conclusion, we have successfully isolated a number of novel nanobodies from a high quality phage display library which have high stability and specific binding affinity to different epitopes of Cry1Ac toxin. Phage display and application of these nanobodies could be a promising approach for the detection of Cry1Ac toxin in agricultural as well as environmental samples. Except for streptavidin-biotin based ELISA, Nbs can be combined with biosensors such as electrochemical impedance spectroscopy and flow injection chemiluminescence, which promise to generate quicker and more sensitive results. By taking advantage of nanobodies, we will put more efforts to develop the immunoassay for sensitive and selective detection in GMOs.

## Supplementary Materials

Supplementary materials can be accessed at: http://www.mdpi.com/2072-6651/6/12/3208/s1.
